# Psoriasis and Risk of Colorectal Cancer: A Nationwide Population-Based Cohort Study in Korea

**DOI:** 10.3390/diseases14060200

**Published:** 2026-06-04

**Authors:** Kyeong Min Han, Dae Myoung Yoo, Ho Suk Kang, Hyo Geun Choi, Joo-Hee Kim, Nan Young Kim, Kyueng-Whan Min, Mi Jung Kwon

**Affiliations:** 1 Hallym Data Science Laboratory, Hallym University College of Medicine, Anyang 14068, Republic of Korea; hankm1130@naver.com (K.M.H.); ydm1285@naver.com (D.M.Y.); 2 Laboratory of Hearing, Balance and Integrated Neuroscience, Hallym University College of Medicine, Anyang 14068, Republic of Korea; hskang76@hallym.or.kr; 3 Division of Gastroenterology, Department of Internal Medicine, Hallym University Sacred Heart Hospital, Hallym University College of Medicine, Anyang 14068, Republic of Korea; 4 Suseo Seoul E.N.T. Clinic, 10, Bamgogae-ro 1-gil, Gangnam-gu, Seoul 06349, Republic of Korea; mdanalytics@naver.com; 5 Division of Pulmonary, Allergy, and Critical Care Medicine, Department of Internal Medicine, Hallym University Sacred Heart Hospital, Hallym University College of Medicine, Anyang 14068, Republic of Korea; luxjhee@hallym.or.kr; 6 Department of Hallym, Institute of Translational Genomics and Bioinformatics, Hallym University Medical Center, Anyang 14068, Republic of Korea; honeyny78@gmail.com; 7 Department of Pathology, Uijeongbu Eulji Medical Center, Eulji University School of Medicine, 712, Dongil-ro, Uijeongbu-si 11759, Republic of Korea; kyueng@gmail.com; 8 Department of Pathology, Hallym University Sacred Heart Hospital, Hallym University College of Medicine, Anyang 14068, Republic of Korea

**Keywords:** psoriasis, colorectal cancer, nationwide cohort, propensity score, overlap weighting, Korean population

## Abstract

Background/Objectives: Psoriasis is a chronic systemic inflammatory disease associated with increased cancer risk; however, its relationship with colorectal cancer (CRC) remains unclear. This question is particularly relevant in Asia, where CRC incidence has been increasing rapidly in recent decades. Methods: We conducted a nationwide longitudinal cohort study using the Korean National Health Insurance Service–National Sample Cohort (2002–2019). Patients with psoriasis were identified using validated ICD-10 codes and matched 1:4 with controls by age, sex, income, and region. Propensity score overlap weighting was applied to achieve covariate balance. Incident CRC was defined using both ICD-10 codes and cancer-specific registration codes. Cox proportional hazards models were used to estimate hazard ratios (HRs) and 95% confidence intervals (CIs). Results: A total of 16,670 patients with psoriasis and 66,680 matched controls were included. During follow-up, the incidence of CRC was comparable between the two groups. In the weighted model, psoriasis was not associated with an increased risk of CRC (aHR = 0.96; 95% CI: 0.83–1.10). Kaplan–Meier analysis showed no significant difference in cumulative incidence (log-rank *p* > 0.05). Subgroup analyses stratified by age, sex, income, region, and comorbidity burden yielded consistent null findings. Conclusions: In this large nationwide cohort, psoriasis was not associated with an increased risk of CRC. These findings suggest that psoriasis does not independently contribute to CRC risk at the population level and does not warrant additional CRC surveillance beyond established screening recommendations, even in a high-burden setting such as Korea.

## 1. Introduction

Psoriasis is a chronic, immune-mediated inflammatory disorder affecting the skin and joints and is increasingly recognized as a systemic disease rather than a condition confined to the skin alone [1,2]. The prevalence of psoriasis varies markedly by ethnicity and geographic region [3]. Previous studies have reported higher prevalence in Western countries, such as the United States (2.2–4.6%) and Germany (6.5%), whereas Asian countries, including China (0.2–1.5%) and Japan (0.29–1.18%), have shown relatively lower rates [4]. Although the standardized prevalence in South Korea has been reported to be low (0.44–0.45%), it has increased steadily in recent years, making the management of associated comorbidities an emerging public health priority [5].

Beyond its cutaneous manifestations, psoriasis has been associated with a broad spectrum of comorbidities, including metabolic syndrome, cardiovascular disease, and inflammatory bowel disease [6], highlighting the systemic consequences of chronic inflammation [2]. This broader disease framework has prompted growing interest in whether psoriasis may also influence long-term cancer risk [7].

Colorectal cancer (CRC) is one of the most common malignancies worldwide and represents a major public health burden [8]. In particular, South Korea is among Asian countries with a high and increasing burden of CRC, ranking among the leading nations in both incidence and mortality as of 2022 [9,10]. Because chronic inflammation is a well-established contributor to colorectal carcinogenesis, psoriasis has been hypothesized to increase CRC risk through shared inflammatory pathways [11,12]. Increased malignancy risk in inflammatory diseases, including psoriasis, systemic lupus erythematosus, and rheumatoid arthritis, has been linked to persistent systemic inflammation [7,12]. Patients with psoriasis also have a substantially higher risk of inflammatory bowel disease, particularly Crohn’s disease [6], and key pro-inflammatory cytokines implicated in psoriasis pathogenesis, including tumor necrosis factor (TNF)-α, interleukin (IL)-17, and IL-23, are also involved in tumor initiation, angiogenesis, and progression [13]. In addition, chronic inflammation-related gut dysbiosis and shared genetic susceptibility loci have been proposed as potential links between psoriasis and CRC [14,15]. These overlapping mechanisms have led to speculation regarding a potential association between psoriasis and CRC [7,12].

However, epidemiologic evidence regarding the association between psoriasis and CRC remains inconsistent, and robust CRC-specific data are still limited, particularly in East Asian populations, including Korea [16,17]. Large population-based cohort studies have primarily evaluated overall cancer risk, with CRC-specific outcomes often underreported [16,17]. For example, a prospective cohort study of U.S. women reported no significant association between psoriasis and CRC (HR = 1.19; 95% CI: 0.76–1.86) [17], whereas a Taiwanese study reported an increased risk (HR = 1.70; 95% CI: 1.01–2.86) [18]. In contrast, studies conducted in Western populations have generally demonstrated nonsignificant associations, with effect estimates close to the null [16,19,20]. Systematic reviews and meta-analyses have reported a modest increase in overall cancer risk among patients with psoriasis, while findings specific to CRC remain inconclusive and highly heterogeneous across studies [21,22,23,24].

Clarifying this relationship has important clinical and public health implications. If psoriasis independently increases CRC risk, more intensive surveillance strategies may be warranted. Conversely, if no meaningful association exists, routine age-appropriate CRC screening may be sufficient without additional disease-specific monitoring. Therefore, the present study aimed to investigate the association between psoriasis and incident CRC using a nationwide population-based cohort in South Korea. To minimize confounding and improve comparability, we applied exact matching combined with propensity score overlap weighting and examined whether the association remained consistent across major demographic and clinical subgroups.

## 2. Materials and Methods

### 2.1. Study Design and Data Source

This population-based longitudinal cohort study utilized data obtained from the Korean National Health Insurance Service–National Sample Cohort (NHIS–NSC), which was established by the National Health Insurance Service (NHIS) in 2002 [25]. The NHIS–NSC was constructed using systematic stratified random sampling and represents approximately 2.2% of the Korean population [25]. Stratification was performed according to age, sex, income status, and eligibility category [25]. After excluding noncitizens and individuals without identifiable income information, the source population comprised approximately 46.6 million individuals [25].

Participants were followed annually from 2002 through 2019 unless censored because of death or emigration. To preserve population representativeness over time, newborn participants were additionally incorporated into the cohort each year. The database includes nationwide administrative claims data generated through Korea’s universal healthcare system, with diagnoses and procedures coded according to the International Classification of Diseases, 10th Revision (ICD-10). Previous validation studies have demonstrated the reliability and representativeness of this cohort database [25].

All data provided by the NHIS were fully anonymized prior to analysis. The study protocol was conducted in accordance with the principles of the Declaration of Helsinki and approved by the Institutional Review Board of Hallym University (IRB No. 2022-10-008). The requirement for informed consent was waived because the study used de-identified secondary data. As the analysis was based exclusively on anonymized administrative claims data, patients and the public were not directly involved in the study design, data analysis, interpretation of results, or dissemination of findings. The study was conducted in accordance with national ethical guidelines for observational research and reported following the Strengthening the Reporting of Observational Studies in Epidemiology (STROBE) guidelines.

### 2.2. Definition of Psoriasis (Exposure)

Patients with psoriasis were defined as those with at least two healthcare visits associated with ICD-10 codes L40.0–L40.9, M07.0–M07.3, or M09.0. This approach was used to improve diagnostic accuracy [26].

### 2.3. Definition of Colorectal Cancer (Outcome)

Incident CRC was defined using both ICD-10 diagnostic codes (C18–C20) and Korean national registration codes for catastrophic illnesses (V193 and V194). These registration codes are assigned exclusively to patients with confirmed cancer diagnoses who are enrolled in the national reimbursement program for serious diseases [27]. The combined use of diagnostic and registration codes has been widely adopted in Korean claims-based studies to improve diagnostic accuracy and minimize outcome misclassification [27].

### 2.4. Matching and Participant Selection

**Figure 1:**
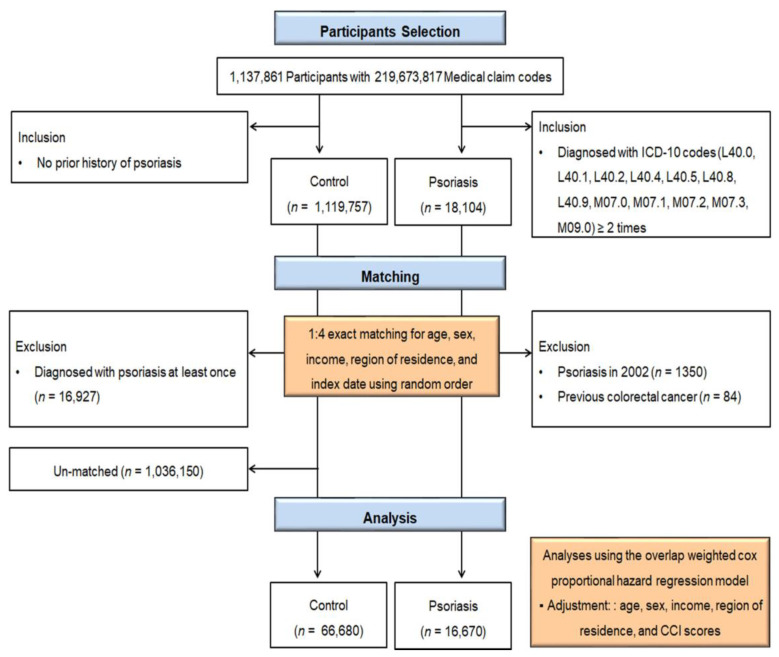
Schematic illustration of the participant selection process in the present study. Among a total of 1,137,861 participants, 16,670 psoriasis participants were matched with 66,680 control participants for age, sex, income, and region of residence. Abbreviations: ICD-10, International Classification of Diseases, 10th Revision; CCI, Charlson Comorbidity Index.

From a total of 1,137,861 participants and 219,673,817 medical claim records collected between 2002 and 2019, we identified 18,104 patients diagnosed with psoriasis, defined as having at least two clinical visits with relevant ICD-10 codes. The potential control pool consisted of 1,119,757 individuals with no history of psoriasis during the study period. Within the psoriasis group, we excluded those diagnosed during the wash-out period in 2002 (*n* = 1350) and those with a pre-existing history of CRC prior to the index date (*n* = 84). In the control group, individuals who received at least one diagnosis of psoriasis (*n* = 16,927) were excluded to ensure the integrity of the cohort. To enhance comparability and mitigate potential confounding, each psoriasis patient was matched to four control participants (1:4 ratio) using systematic stratified random sampling based on age, sex, income level, and region of residence. The index date was defined as the date of the initial treatment for each psoriasis patient, and the same date was assigned to the corresponding matched controls to ensure temporal alignment. During the matching procedure, 1,036,150 unmatched control participants were excluded. Ultimately, 16,670 psoriasis patients were matched with 66,680 control participants (Figure 1).

Participants were followed from their respective index dates until the earliest occurrence of incident CRC, death, or 31 December 2019. Because the study incorporated eligible incident CRC cases and their matched controls within the health screening cohort, the sample size was determined by data availability rather than a priori power calculation.

### 2.5. Covariates

To account for potential confounding factors, participants were classified into 18 distinct age categories, each spanning a 5-year increment. Socioeconomic status was represented by income levels, divided into quintiles ranging from the lowest (Level 1) to the highest (Level 5). Based on 16 administrative districts, residential areas were dichotomized into urban and rural regions. Furthermore, the overall comorbidity status of each participant was quantified using the Charlson comorbidity index (CCI). This index assigns weights to 17 medical conditions, resulting in a composite score between 0 and 29, to adjust for the baseline health status of the study population [28]. CCI scores were derived by mapping ICD-10 diagnostic codes to predefined comorbidity categories based on established algorithms validated for use in administrative health data [28]. These algorithms have been widely used in epidemiological studies utilizing Korean National Health Insurance data [29].

### 2.6. Statistical Analyses

Propensity score (PS) overlap weighting was utilized to achieve baseline balance between the psoriasis and control groups while maximizing statistical efficiency [30]. Propensity scores were estimated using multivariable logistic regression, incorporating all baseline covariates used in the matching process, including age, sex, income level, region of residence, and CCI scores [30]. This approach ensured consistency between the matching and weighting procedures and minimized residual confounding [30]. Overlap weights were calculated as 1—PS for the psoriasis group and PS for the control group, constraining weights between 0 and 1 and improving covariate balance and estimation efficiency [31].

The adequacy of covariate balance after weighting was assessed using standardized mean differences (SMD), with an absolute SMD < 0.20 considered indicative of adequate balance [31]. Crude incidence rates for CRC were expressed as the number of events per 1000 person-years. Kaplan–Meier survival curves were generated to estimate cumulative incidence, and differences between groups were compared using the log-rank test.

The association between psoriasis and CRC risk was evaluated using Cox proportional hazards regression models with overlap weighting. Hazard ratios (HRs) and 95% confidence intervals (CIs) were reported for both crude and weighted models. The proportional hazards assumption was assessed using log-minus-log plots and graphical inspection.

Subgroup analyses were conducted according to age (<50 vs. ≥50 years), sex, comorbidity burden, income level, and region of residence. All statistical analyses were performed using SAS software (version 9.4; SAS Institute Inc., Cary, NC, USA), and two-sided *p*-values < 0.05 were considered statistically significant.

## 3. Results

### 3.1. Baseline Characteristics

Table 1 summarizes the baseline characteristics of the 16,670 patients in the psoriasis group and their 66,680 matched controls, following a 1:4 allocation based on age, sex, income, and residential area. Immediately after the exact matching procedure, all evaluated covariates achieved an SMD of 0.00, demonstrating an exceptionally high degree of parity between the two cohorts. The subsequent application of overlap weighting further ensured that the SMD for all covariates remained at 0, confirming an ideal balance across all demographic and clinical variables. These findings indicate that potential confounding factors were effectively minimized, providing a robust foundation for the comparative analysis of CRC incidence.

### 3.2. Association Between Psoriasis and Colorectal Cancer

As summarized in Table 2, the total follow-up duration was 130,506 person-years in the psoriasis group and 520,363 person-years in the control group. Median follow-up durations were 8.2 years (interquartile range [IQR], 5.1–12.4 years) in the psoriasis group and 8.3 years (IQR, 5.0–12.5 years) in the control group.

Patients with psoriasis exhibited a similar risk of CRC compared to the control group in both crude and adjusted analyses; however, no statistically significant association was observed. In the crude model, psoriasis was associated with a negligible 1% reduction in CRC risk (HR = 0.99; 95% CI: 0.83–1.18; *p* = 0.94). Following propensity score overlap weighting to adjust for comprehensive confounders, the adjusted HR (aHR) was 0.96 (95% CI: 0.83–1.10; *p* = 0.54), remaining statistically nonsignificant.

**Figure 2:**
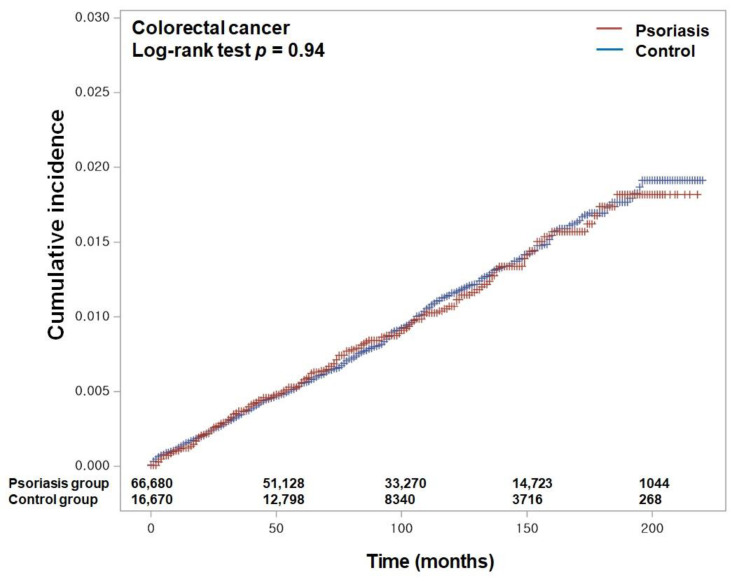
Kaplan–Meier curves for cumulative incidence of colorectal cancer in patients with psoriasis and matched controls. No significant difference in colorectal cancer incidence was observed between the two groups (log-rank *p* = 0.94).

Kaplan–Meier analysis with the log-rank test further confirmed that the cumulative incidence of CRC did not differ significantly between the two cohorts (*p* = 0.94; Figure 2). The survival curves remained closely aligned and overlapped throughout the follow-up period, indicating no temporal divergence in CRC risk between psoriasis patients and the matched controls.

### 3.3. Subgroup Analyses

Subgroup analyses based on the overlap weighting-adjusted model are presented in Table 2. Across all stratified analyses, no statistically significant association between psoriasis and CRC risk was identified, with HR consistently observed near 1.0.

Specifically, when stratified by age, the risk of CRC did not show significant differences in either the age < 50 years old group (aHR = 1.04; 95% CI: 0.73–1.48; *p* = 0.81) or the age ≥ 50 years old group (aHR = 0.93; 95% CI: 0.80–1.09; *p* = 0.37). Regarding sex, the association remained nonsignificant for both male (aHR = 0.95; 95% CI: 0.80–1.12; *p* = 0.55) and female (aHR = 0.98; 95% CI: 0.77–1.25; *p* = 0.85) participants.

Further analyses by socioeconomic status and residence also yielded nonsignificant findings. For instance, the risk was similar between the low-income group and the high-income group, with no significant divergence from the control group. Similarly, those categorized as urban residents (aHR = 0.99; 95% CI: 0.81–1.22; *p* = 0.95) and rural residents (aHR = 0.92; 95% CI: 0.76–1.11; *p* = 0.39) showed no statistical association with increased CRC incidence.

Finally, the comorbidity burden did not significantly influence the results. The risk remained nonsignificant across all categories, including CCI scores = 0 (aHR = 0.94; 95% CI: 0.59–1.47; *p* = 0.77), CCI scores = 1, and CCI scores ≥ 2.

## 4. Discussion

In this nationwide longitudinal cohort study based on a representative Korean population, psoriasis was not associated with an increased risk of incident CRC. After exact matching and additional adjustment using propensity score overlap weighting, the hazard of CRC among patients with psoriasis remained comparable to that of matched controls (aHR = 0.96; 95% CI: 0.83–1.10). This null association was consistently observed across all subgroup analyses, and cumulative incidence curves remained closely overlapping throughout follow-up. Taken together, these findings suggest that psoriasis is unlikely to independently increase CRC risk, even in a setting such as South Korea where the burden of CRC is high [10].

Our findings are consistent with prior studies suggesting that the association between psoriasis and CRC is weak or nonsignificant across populations. Although some cohort studies have reported an increased risk [18,32], most large-scale analyses have shown effect estimates clustered around the null value [19,21,23,24]. For example, a nationwide cohort study from Germany reported a nonsignificant association (OR = 1.11; 95% CI: 0.97–1.27) [20], and a prospective cohort study of U.S. women similarly found no significant increase in CRC risk (HR = 1.02; 95% CI: 0.71–1.73) [17].

Meta-analytic studies have suggested a modestly increased CRC risk (relative risk approximately 1.3), with substantial between-study heterogeneity [21,22]. A recent meta-analysis focusing specifically on CRC reported an elevated risk (HR = 1.22; 95% CI: 1.11–1.34), with variation across regions and sex [22]. Taken together, these findings indicate that any potential association between psoriasis and CRC may be modest and not consistently observed across different study settings. The heterogeneity observed across prior studies may be attributable to regional differences in genetic background, environmental exposures, healthcare access, and cancer screening practices [33]. In particular, variations in the availability and uptake of organized screening programs may substantially influence cancer detection and reported incidence across populations [33]. In South Korea, where the burden of CRC is high and a nationwide screening program is widely implemented [34], these factors may contribute to a more uniform detection environment and a more accurate estimation of the underlying epidemiologic association.

Evidence from Korea has similarly suggested that psoriasis may not be associated with CRC [23,24,35]. Previous nationwide cohort studies reported slightly increased risks of overall malignancy (HRs approximately 1.06–1.10), but these were largely driven by specific cancers such as lymphoma, non-melanoma skin cancer, lung cancer, or gastric cancer rather than CRC [23,24,35]. Our findings extend these studies [23,24,35] by focusing specifically on incident CRC and by applying rigorous methodological approaches, including exact matching and propensity score overlap weighting, to achieve excellent covariate balance. These methodological strengths reduce potential confounding and strengthen the validity of the observed null association. The consistency of our findings across all demographic and clinical strata further supports the conclusion that psoriasis is not an independent risk factor for CRC in the Korean population.

Several explanations may account for the absence of an association despite biological plausibility. First, although psoriasis involves chronic systemic inflammation [7,12], the magnitude or pattern of inflammatory activity may not be sufficient to independently increase CRC risk at the population level. Second, psoriasis is a heterogeneous condition, encompassing a wide spectrum of disease duration, severity, and treatment exposure [1,19,23,36]. In chronic inflammatory diseases, cancer risk may evolve over prolonged periods, and it is possible that any potential increase in CRC risk may be confined to specific subgroups of patients, such as those with long-standing or more severe disease [19,21,37]. However, these factors could not be adequately evaluated in the present study due to the inherent limitations of administrative claims data [26]. In addition, unmeasured lifestyle factors—including smoking, alcohol consumption, diet, and obesity—may influence CRC risk and contribute to residual confounding [38]. Although these variables were not available in the dataset, their potential impact should be considered when interpreting the findings.

Third, evolving treatment patterns may also play an important role. Contemporary management of psoriasis increasingly involves biologic therapies targeting the IL-17 and IL-23 pathways [36,39], which may modify the systemic inflammatory milieu and potentially attenuate inflammation-driven carcinogenic processes [7,40]. Emerging evidence suggests that these targeted therapies may be associated with a lower risk of certain malignancies compared with conventional systemic treatments [41], although the available findings remain heterogeneous. In addition, some studies have reported that systemic phototherapy and oral systemic therapies were not associated with a significantly increased overall cancer risk [18]. Collectively, these treatment-related factors may partly contribute to the absence of an observed increase in CRC risk at the population level in the present study [18].

In addition, the Korean healthcare system provides broad access to medical services and operates a well-established national cancer screening program [25]. In many observational settings, patients with chronic diseases such as psoriasis may have more frequent healthcare contact, leading to increased opportunities for cancer detection and potentially inflating observed cancer risk through detection bias [42]. In Korea, the widespread availability of standardized screening likely reduces disparities in diagnostic opportunity, allowing for a more accurate estimation of the underlying epidemiologic association [10,25,42]. The closely overlapping Kaplan–Meier curves in our study further support the absence of meaningful differences in CRC incidence over time.

From a clinical and public health perspective, these findings have important implications. Given the high prevalence of psoriasis, even a modest increase in CRC risk could have meaningful consequences for screening strategies [20]. However, our results do not support modification of current CRC screening practices or the need for additional CRC surveillance solely on the basis of psoriasis [24]. These findings are consistent with current clinical practice recommendations, which do not advocate intensified cancer screening in patients with psoriasis in the absence of established risk factors.

In the context of South Korea, where CRC incidence and mortality remain high and organized screening programs are widely implemented [10,34], our findings provide reassurance that standard population-based screening strategies are likely sufficient for patients with psoriasis. While caution is warranted in extrapolating these findings, similar implications may be relevant to other Asian countries experiencing a rising CRC burden, particularly those with comparable healthcare access and screening systems.

It should also be noted that psoriasis is a heterogeneous disease, and prior studies have suggested that cancer risk may differ according to disease severity or duration. Because our analysis did not include detailed clinical information on psoriasis severity or treatment exposure, the present findings reflect the overall population-level risk rather than risk within specific high-risk subgroups. Therefore, the possibility of differential risk among patients with severe or longstanding psoriasis cannot be excluded and warrants further investigation in future studies.

This study has several important strengths. First, it was based on a large, nationally representative longitudinal cohort with long-term follow-up, enabling the evaluation of incident CRC in a real-world population setting [25]. Importantly, the median follow-up durations were 8.2 years (IQR, 5.1–12.4 years) in the psoriasis group and 8.3 years (IQR, 5.0–12.5 years) in the control group, corresponding to more than 650,000 person-years of observation overall. These extended follow-up periods were likely sufficient to capture incident CRC events, considering the prolonged natural history of colorectal carcinogenesis through the adenoma–carcinoma sequence, which may involve dwelling times of approximately 16 years for tubular adenoma, 9 years for tubulovillous adenoma, and 4 years for villous adenoma [43]. This extended longitudinal follow-up strengthens the robustness of the null association observed in the present study. Second, the combined use of 1:4 exact matching and propensity score overlap weighting achieved excellent balance across measured baseline covariates (all SMDs = 0.00), thereby minimizing confounding and enhancing internal validity [31,44]. Third, the outcome definition incorporated both ICD-10 diagnostic codes and national cancer-specific registration codes, which likely improved diagnostic accuracy and reduced outcome misclassification [45]. Finally, the consistency of findings across multiple subgroup analyses supports the robustness of the primary results and suggests that the observed null association is unlikely to be explained by methodological limitations or insufficient statistical power [46].

Several limitations should be acknowledged. First, because this study relied on administrative claims data, we were unable to account for important clinical and lifestyle factors, including psoriasis severity, disease duration prior to cohort entry, body mass index, smoking, alcohol consumption, dietary habits, family history of CRC, colonoscopy history, and detailed medication exposure. Residual confounding from these unmeasured variables therefore remains possible. In particular, the inability to distinguish between mild and severe psoriasis may have obscured potential heterogeneity in CRC risk across disease subtypes. However, our findings remain informative at the population level, as they reflect the overall risk of CRC across the full spectrum of patients with psoriasis. Second, although the claims-based definition of psoriasis requiring at least two healthcare visits likely improved diagnostic validity, some degree of misclassification cannot be excluded. Finally, as with all observational studies, causal inferences cannot be established, and the findings should be interpreted as associations rather than evidence of a causal relationship.

## 5. Conclusions

In this large, nationwide population-based cohort, psoriasis was not associated with an increased risk of incident CRC in South Korea. The effect estimate remained close to the null after rigorous control of measured confounders, and the findings were consistent across all examined subgroups. These findings suggest that psoriasis is unlikely to independently increase CRC risk at the population level.

From a clinical perspective, our findings support the use of standard, age-appropriate CRC screening strategies without additional surveillance solely on the basis of psoriasis. In the context of South Korea, where the burden of CRC is high and organized screening programs are widely implemented [10,34], these findings provide reassurance regarding current screening practices. While the results may have implications for other Asian populations with a similarly high CRC burden, further studies are warranted to evaluate potential risk heterogeneity according to disease severity, duration, and treatment exposure.

## Figures and Tables

**Table 1:** Summary of baseline features of the participants.

Characteristics	Before PS Overlap Weighting Adjustment			After PS Overlap Weighting Adjustment		
	Psoriasis	Control	SMD	Psoriasis	Control	SMD
Age, *n* (%)			0.00			0.00
0–4	173 (1.04)	692 (1.04)		138 (1.04)	138 (1.04)	
5–9	292 (1.75)	1168 (1.75)		234 (1.75)	234 (1.75)	
10–14	438 (2.63)	1752 (2.63)		350 (2.63)	350 (2.63)	
15–19	709 (4.25)	2836 (4.25)		567 (4.26)	567 (4.26)	
20–24	895 (5.37)	3580 (5.37)		716 (5.37)	716 (5.37)	
25–29	1154 (6.92)	4616 (6.92)		923 (6.93)	923 (6.93)	
30–34	1231 (7.38)	4924 (7.38)		984 (7.39)	984 (7.39)	
35–39	1323 (7.94)	5292 (7.94)		1058 (7.94)	1058 (7.94)	
40–44	1333 (8.00)	5332 (8.00)		1066 (8.00)	1066 (8.00)	
45–49	1505 (9.03)	6020 (9.03)		1203 (9.03)	1203 (9.03)	
50–54	1579 (9.47)	6316 (9.47)		1262 (9.47)	1262 (9.47)	
55–59	1450 (8.70)	5800 (8.70)		1158 (8.69)	1158 (8.69)	
60–64	1253 (7.52)	5012 (7.52)		1002 (7.52)	1002 (7.52)	
65–69	1158 (6.95)	4632 (6.95)		926 (6.95)	926 (6.95)	
70–74	1004 (6.02)	4016 (6.02)		802 (6.02)	802 (6.02)	
75–79	672 (4.03)	2688 (4.03)		537 (4.03)	537 (4.03)	
80–84	340 (2.04)	1360 (2.04)		272 (2.04)	272 (2.04)	
85+	161 (0.97)	644 (0.97)		128 (0.96)	128 (0.96)	
Sex, *n* (%)			0.00			0.00
Male	9014 (54.07)	36,056 (54.07)		7205 (54.07)	7206 (54.07)	
Female	7656 (45.93)	30,624 (45.93)		6120 (45.93)	6120 (45.93)	
Income, *n* (%)			0.00			0.00
1 (lowest)	3213 (19.27)	12,852 (19.27)		2568 (19.27)	2568 (19.27)	
2	2399 (14.39)	9596 (14.39)		1918 (14.39)	1918 (14.39)	
3	2918 (17.50)	11,672 (17.50)		2333 (17.51)	2333 (17.51)	
4	3680 (22.08)	14,720 (22.08)		2942 (22.07)	2942 (22.07)	
5 (highest)	4460 (26.75)	17,840 (26.75)		3565 (26.75)	3565 (26.75)	
Region of residence, *n* (%)			0.00			0.00
Urban	7454 (44.72)	29,816 (44.72)		5959 (44.72)	5959 (44.72)	
Rural	9216 (55.28)	36,864 (55.28)		7367 (55.28)	7367 (55.28)	
CCI score, mean ± SD	0.66 ± 1.41	0.58 ± 1.33	0.06	0.65 ± 1.24	0.65 ± 1.24	0.00
CRC, *n* (%)	157 (0.94)	630 (0.94)	0.00	122 (0.92)	140 (1.05)	0.01

Abbreviations: PS, propensity score; CCI, Charlson Comorbidity Index; SMD, standardized mean differences; SD, standard deviation; CRC, colorectal cancer.

**Table 2:** Incidence rates and hazard ratios for colorectal cancer (CRC) according to psoriasis history.

	N of Event/ N of Total (%)	Follow-Up Duration (PY)	IR per 1000 (PY)	IRD (95% CI)	Hazard Ratios for CRC			
					Crude	*p*	Adjusted Model with OW †	*p*
Total participants (*n* = 83,350)								
Psoriasis	157/16,670 (0.94)	130,506	1.20	−0.01 (−0.22–0.20)	0.99 (0.83–1.18)	0.94	0.96 (0.83–1.1)	0.54
Control	630/66,680 (0.94)	520,363	1.21		1		1	
Age < 50 years old (*n* = 45,265)								
Psoriasis	26/9053 (0.29)	76,897	0.34	0.04 (−0.10–0.18)	1.14 (0.74–1.76)	0.55	1.04 (0.73–1.48)	0.81
Control	91/36,212 (0.25)	307,278	0.30		1		1	
Age ≥ 50 years old (*n* = 38,085)								
Psoriasis	131/7617 (1.72)	53,609	2.44	−0.09 (−0.56–0.39)	0.97 (0.80–1.17)	0.73	0.93 (0.80–1.09)	0.37
Control	539/30,468 (1.77)	213,085	2.53		1		1	
Male (*n* = 45,070)								
Psoriasis	106/9014 (1.18)	68,673	1.54	−0.02 (−0.35–0.31)	0.99 (0.80–1.22)	0.92	0.95 (0.80–1.12)	0.55
Control	427/36,056 (1.18)	273,731	1.56		1		1	
Female (*n* = 38,280)								
Psoriasis	51/7656 (0.67)	61,833	0.82	0.00 (−0.25–0.25)	1.00 (0.74–1.36)	0.99	0.98 (0.77–1.25)	0.85
Control	203/30,624 (0.66)	246,632	0.82		1		1	
Low income group (*n* = 42,650)								
Psoriasis	75/8530 (0.88)	66,984	1.12	−0.05 (−0.34–0.24)	0.96 (0.74–1.23)	0.73	0.98 (0.80–1.20)	0.85
Control	312/34,120 (0.91)	266,383	1.17		1		1	
High income group (*n* = 40,700)								
Psoriasis	82/8140 (1.01)	63,522	1.29	0.04 (−0.27–0.35)	1.03 (0.81–1.31)	0.81	0.96 (0.79–1.16)	0.66
Control	318/32,560 (0.98)	253,980	1.25		1		1	
Urban resident (*n* = 37,270)								
Psoriasis	72/7454 (0.97)	59,374	1.21	0.06 (−0.24–0.37)	1.06 (0.81–1.37)	0.68	0.99 (0.81–1.22)	0.95
Control	272/29,816 (0.91)	236,844	1.15		1		1	
Rural resident (*n* = 46,080)								
Psoriasis	85/9216 (0.92)	71,132	1.19	−0.07 (−0.36–0.22)	0.95 (0.75–1.20)	0.65	0.92 (0.76–1.11)	0.39
Control	358/36,864 (0.97)	283,519	1.26		1		1	
CCI scores = 0 (*n* = 61,495)								
Psoriasis	14/11,832 (0.12)	94,208	0.15	−0.01 (−0.10–0.08)	0.93 (0.52–1.65)	0.80	0.94 (0.59–1.47)	0.77
Control	63/49,663 (0.13)	392,797	0.16		1		1	
CCI scores = 1 (*n* = 10,256)								
Psoriasis	7/2239 (0.31)	17,282	0.41	0.09 (−0.22–0.40)	1.27 (0.54–3.01)	0.58	1.33 (0.63–2.79)	0.45
Control	20/8017 (0.25)	62,697	0.32		1		1	
CCI scores ≥ 2 (*n* = 11,599)								
Psoriasis	136/2599 (5.23)	19,016	7.15	−1.28 (−2.74–0.18)	0.85 (0.70–1.02)	0.08	0.87 (0.75–1.01)	0.07
Control	547/9000 (6.08)	64,869	8.43		1		1	

Abbreviation: CCI, Charlson Comorbidity Index; IR, incidence rate; IRD, incidence rate difference; PY, person-years; CI, confidence interval; OW, overlap weighting. Significance at *p* < 0.05. † Adjusted for age, sex, income, region of residence, and CCI scores.

## Data Availability

All data are available from the database of National Health Insurance Sharing Service (NHISS) https://nhiss.nhis.or.kr/ (accessed on 1 October 2024). NHISS allows access to all of this data for any researcher who promises to follow the research ethics at some processing charge. If you want to access the data of this article, you can download it from the website after promising to follow the research ethics.
